# Infant Formula Crisis in China: A Cohort Study in Sichuan Province

**Published:** 2015-03

**Authors:** Li Tang, Colin W. Binns, Andy H. Lee

**Affiliations:** School of Public Health, Curtin University, Perth, Australia

**Keywords:** Infant formula, Insufficient breastmilk, Melamine scandal, Mothers' opinion, China

## Abstract

China has become the largest market of infant formula in the world. The consumption of infant formula is widespread across the country. This study investigated the opinions of Chinese mothers on infant formula. A prospective cohort study (n=695) was undertaken in 2011 in Sichuan province of China two years after the melamine scandal. Infant-feeding practices and mothers’ opinions on infant formula-use were documented in detail. A total of 674 mothers (97%) had initiated breastfeeding by discharge. Of the 21 mothers who did not commence breastfeeding, 13 made a decision to exclusively feed their babies with infant formula because of hepatitis B virus infection. Nearly 70% of newborns received infant formula as their first feed, and the prevalence increased to 88% within one month. Having insufficient breastmilk was perceived by the majority (77%) of mothers as the reason behind infant formula feeding. About half (46%) of the mothers agreed with or were ambivalent that infant formula feeding does not reduce their breastmilk production. More than one-third (38%) of women thought that formulafed infants sleep longer at night than those who are breastfed. In addition, this perception was positively associated with the use of formula within one month postpartum (p=0.003). In conclusion, mothers’ opinions appear to influence the use of infant formula in China. There is a need for further education on breastfeeding and infant-feeding options to maintain and improve breastfeeding outcomes in China.

## INTRODUCTION

Breastfeeding is the most appropriate feeding method for infants and young children (1). It is only half a century ago that breastfeeding was the norm in China; almost all infants were breastfed with a median breastfeeding duration of 12 months (2). For the majority of the Chinese population, no infant formula or other alternative was available. Today, China has become the largest market of infant formula in the world. A recent market report showed that the Chinese market is growing rapidly at an annual rate of 20% and accounts for nearly one-quarter of the world's infant formula market (3). Less than 30% infants younger than 6 months had been exclusively breastfed as found in a large survey in central and western China. Using formula was one of the most common reasons for non-exclusive breastfeeding during the first six months (4). The rate of infant formula-use by newborns was higher in east coastal areas. A recent cohort study in Zhejiang province of China found that over half of the infants had already consumed, or were consuming, some infant formula by one month postpartum, and the proportion reached 98% by six months (5).

Since the melamine scandal in 2008, many Chinese parents have become concerned about the safety of domestically-produced infant formula. Melamine, an industrial chemical, was added to adulterate processed milk to give the appearance of attaining normal levels of protein. According to the World Health Organization (WHO), the melamine-contaminated milk powder and related dairy products caused the deaths of at least six infants, and more than 51,900 infants and young children were hospitalized with renal problems (6). Although measures, such as the recall of potentially-contaminated products and severe punishments for the manufacturers at fault, were taken by the Chinese Government immediately after the melamine scandal, tonnes of tainted milk powder were still illegally resold two years later (7). In 2011, the concerns about safety of formula milk were further worsened by some manufacturers’ practice of adding leather-hydrolyzed protein, derived from leather scraps, in milk products in China (8).

After a series of scandals on contaminated milk and added pressures from the one child policy, Chinese parents have lost confidence in domestically-manufactured infant formula. With the rapidly-increasing demand for safe formula, panic buying from overseas becomes evident, to the extent of causing shortages of infant formula in neighbouring countries. For example, mainland visitors and parallel traders had bought up large quantities of infant formula in Hong Kong and taken them across the border since the melamine scandal (9). In response to complaints from local parents about the difficulties of purchasing infant formula, the Hong Kong Government recently introduced a basket of actions, such as limiting the amount being taken out of the city to 1.8 kg (2 cans) per person (10). Unavailability of several popular brands of infant formula was also reported in Australia. Some pharmacies and supermarkets now restrict the sale of formula per time, which is believed to have resulted from Chinese customers purchasing infant formula in bulk quantities and shipping them back to China (11,12). More recently, however, the potential botulism contamination of imported formula may have undermined the Chinese parents’ confidence in dairy products from overseas and worsened their concerns about the safety of infant formula (13).

A number of articles appeared in the lay press on the infant formula crisis in China and the way Chinese parents are purchasing formula worldwide. The present study provides the first academic report investigating the opinions of Chinese mothers on infant formula-use.

## MATERIALS AND METHODS

In 2010, two years after the melamine scandal, a prospective cohort study of infant-feeding was undertaken in Jiangyou, Sichuan province of China. Sichuan is one of the most populous provinces in China that sustained severe earthquakes in recent years. Jiangyou is a city with a population of 880,000, located 160 km north of the provincial capital Chengdu. Between March and November 2010, mothers who delivered at four general hospitals and three township health centres in Jiangyou were recruited to participate in a face-to-face interview before discharge. The four hospitals covered the entire catchment region while the three health centres were randomly selected from rural areas. All seven institutions are certified to be ‘baby-friendly’. Follow-up interviews were then carried out at 1, 3, and 6 months postpartum by telephone. Information on infant-feeding practices as well as mothers’ opinions on infant formula was collected. The structured questionnaire included questions on attitudes towards breastfeeding and the use of infant formula. Data were entered and analyzed using Statistical Package for the Social Sciences (SPSS) (version 18.0) (14). Frequencies were used for describing the rate of breastfeeding initiation, distribution of infants’ first feeds, and mothers’ opinions on infant formula. Chi-square tests were applied to investigate the associations between mothers’ opinions on infant formula and the use of formula within one month postpartum.

This study adopted breastfeeding definitions recommended by the WHO (15). Exclusively-breastfed infants are those who receive only breastmilk without any liquid or solid (except vitamins, mineral supplements, or medications). ‘Full breastfeeding’ is defined as breastmilk being the infant's main source of nourishment. A fully-breastfed infant may have received only breastmilk without other liquids or solids (except vitamins, mineral supplements, or medications), or breastmilk and water, water-based drinks, fruit juice, and oral rehydration salts but no breastmilk substitutes or solids. The term ‘prelacteal feeds’ is defined as any feeds given before the onset of copious breastmilk secretion (16).

The study protocol was approved by the participating facilities and the Human Research Ethics Committee of Curtin University (approval number HR169/2009). An information sheet explaining the project was given and read to the women before obtaining their written consent. All participants were informed about their right to withdraw without prejudice.

## RESULTS

A total of 695 mothers (response rate 96%) consented to participate in this study. Table 1 presents the characteristics of the participants. The 695 women were aged between 18 and 44 years (median 24 years), and the caesarean section rate was 71.4%. Approximately 80% of mothers were delivering their first baby, and birthweights of the great majority (94%) of infants fell within the normal range.

**Table 1.** Characteristics of the participants (N=695)

Variable	No. (%)
Maternal age (completed years)	
<25	397 (57.1)
25-29	178 (25.6)
30-34	78 (11.2)
≥35	42 (6.0)
Marital status	
Married	693 (99.8)
Never married/Divorced/Separated	2 (0.2)
Maternal education (years)	
≤9	380 (54.7)
10-12	233 (33.5)
>12	82 (11.8)
Maternal employment	
Labour job	289 (41.6)
Office job	189 (27.2)
No job	217 (31.2)
Gestation (weeks)	
<37	11 (1.6)
≥37	681 (98.0)
Unknown	3 (0.4)
Method of delivery	
Vaginal delivery	199 (28.6)
Caesarean section	496 (71.4)
Parity	
Primiparous	555 (79.9)
Multiparous	140 (20.1)
Infant's gender	
Male	341 (49.1)
Female	354 (50.9)
Infant's birthweight (g)	
<2,500	12 (1.7)
2,500-3,999	653 (94.0)
≥4,000	30 (4.3)

By discharge, 674 (97%) participants had initiated breastfeeding or had tried to breastfed. Only 21 (3%) mothers made a decision to exclusively feed their babies with infant formula from the start. None of these mothers reported “formula was better than or as good as breastmilk” or equivalent wording as their reason. Thirteen (2%) of the mothers did not commence breastfeeding because of hepatitis B virus infection. Mother-to-child transmission was their main concern for not breastfeeding.

All mothers agreed that breastmilk is better than infant formula, at least when interviewed at discharge. However, despite the fact that only a small proportion (3%) of mothers chose to use formula exclusively, prelacteal feeding of infants was predominantly done by infant formula. Only 6.8% of babies received colostrum or breastmilk as their first feed whereas infant formula, plain water, and other fluids accounted for 67.9%, 22.7%, and 2.6% respectively. Less than 10% of mothers initiated breastfeeding within one hour after birth, and the majority (75.9%) of them commenced after 24 hours. Before discharge, nearly 40% of our mothers received free samples of infant formula. By one month, infant formula had been given to 88.3% of the babies.

The 595 mothers who remained in the cohort at 6 months postpartum were asked three questions concerning infant formula. The response options were ‘true’, ‘false’ and ‘don't know’. The statements and results are presented in Table 2. The majority (77%) of Chinese mothers believed that insufficient breastmilk is the reason behind infant formula feeding. Approximately half (46%) of the mothers agreed with or were ambivalent that feeding a one-month old baby with formula does not reduce breastmilk production. More than one-third (38%) of women thought that formulafed infants sleep longer at night than those who are breastfed. Indeed, this perception was positively associated with the use of formula within one month postpartum (p=0.003).

## DISCUSSION

This study found that mothers' opinions contribute to the high consumption of infant formula in China. The observed caesarean section rate of 71.4% was higher than previously-reported rate of 46.2% in 2008 (17) but comparable with rates documented in other parts of China (18,19). To curtail the use of infant formula, several misunderstandings on breastfeeding should be rectified. First, with a high rate of chronic hepatitis B infection in China, infected mothers must be informed and educated during antenatal session that breastfeeding remains the ideal method for feeding their newborns unless nipples are damaged or bleeding (20). It is necessary to stress that breastfeeding will not elevate the risk of hepatitis B infection to the infant (21,22).

Second, any prelacteal feeds should be avoided. Instead, mothers should be encouraged to initiate breastfeeding as soon as practicable, preferably within the first 30-60 minutes after birth. The high rates of prelacteal feeding and delayed breastfeeding initiation have been reported in previous studies (16,23). This phenomenon may be attributed to the belief that breastfeeding should wait until the breasts become engorged, which means deferring breastfeeding until the second day postpartum or later (24). The determinants of early breastfeeding initiation within the first hour have been reported elsewhere (25). In future educational programme, there is a need to emphasize the benefits of starting breastfeeding within one hour after birth and the advantages of exclusive breastfeeding for the first six months. Also, all maternal healthcare practitioners should be educated and trained to assist mothers to initiate breastfeeding early.

**Table 2.** Mothers’ opinions on infant formula feeding (N=595)

Statement	True	False	Don't know
There are lots of women who need to give their babies formula because they can't make enough milk	459 (77.1%)	93 (15.6%)	43 (7.3%)
Feeding formula to a one-month old baby will not reduce the amount of milk produced by the mother	123 (20.7%)	322 (54.1%)	150 (25.2%)
Formulafed babies sleep longer at night	228 (38.3%)	159 (26.7%)	208 (35.0%)

In addition, almost eight in 10 Chinese mothers believed in the need for infant formula because of insufficient breastmilk. Actually, according to previous physiological studies, only 1% to 5% of women have genuine problems with milk supply while the vast majority can produce more than enough milk for their newborns (26). It is, thus, important for health educators in China to break the myth by strengthening the notion that sucking the breast can best stimulate milk production. Feeding infants with formula, especially in the early months, is most likely to decrease the breastmilk supply. More importantly, new mothers and their babies need to be helped soon after delivery to establish an effective latch to ensure sufficient breastmilk, consequently preventing the use of formula.

Approximately 40% of Chinese mothers thought that formulafed babies sleep longer than those breastfed at night. Moreover, significant association was observed between this perception and formula-use within the first month. We found that infants who were exclusively fed with formula woke up less frequently than fully-breastfed infants during the night hours (10.00 pm–6.00 am) at 1, 3, and 6 months of age. As frequent nursing at night and lack of sleep may lead to breastfeeding cessation, providing new parents with instructions and techniques to enable their breastfed babies to sleep better throughout the night will minimize the reliance on formula.

The potential health risks of consuming formula, even safe formula of good quality, compared to breastfeeding, should be discussed with the Chinese parents. The rapid weight gain during infancy is one of the adverse effects of infant formula. ‘Rapid weight gain’ and ‘the heavier the better’ are equated with a healthier baby in traditional Chinese beliefs (18). It is important to provide the Chinese parents with educational messages on the detrimental impact of gaining weight too fast.

Last but not the least, the distribution of formula samples at hospitals, even baby-friendly hospitals, has been reported by the media in different parts of the country (27,28). It has been shown that distribution of formula samples may decrease breastfeeding rates and cause earlier cessation of breastfeeding (29). China needs to adopt stronger regulations and enforcement on the marketing of infant formula. Punishments for formula companies which violated such rules should not be limited to warnings and fines. A recent report by Save the Children showed substantial distinctions in the retail value sales of formula between China and India. India, with stricter marketing legislation and enforcement, kept its sales of formula to remain at a much lower level throughout 2002 to 2008 (30).

### Limitations

Our participants were recruited from one location in Sichuan province. China is a vast country with diverse regional characteristics; so, infant-feeding practices and mothers’ perceptions may differ between areas. For full comprehension of the reasons behind the widespread use of infant formula, further replications and new studies in different parts of China are recommended.

### Conclusions

This study suggests that mothers’ opinions contribute to the prevalent consumption of infant formula in China. To discourage the use of infant formula and promote breastfeeding in China, the combined efforts of health workers and community services are required. There is a need for the Chinese Government to consider adopting stricter regulations and enforcement for the marketing of infant formula, which will help resolve the current infant-feeding crisis in China.

## ACKNOWLEDGEMENTS

The authors thank the mothers for their time and participation in the study and the hospital staff who assisted in the recruitment. No funding was received.

**Conflict of interest**: Authors declare that there are no conflicts of interest.
